# Synthesis of a [^18^F]F Estradiol Derivative via Click Chemistry Using an Automated Synthesis Module: In Vitro Evaluation as Potential Radiopharmaceutical for Breast Cancer Imaging

**DOI:** 10.3390/ph17030388

**Published:** 2024-03-18

**Authors:** María Emilia Tejería, María Pía Pereira, Juan Pablo Gambini, Pablo Duarte, Javier Gabriel Giglio, Ana María Rey

**Affiliations:** 1 Área Radioquímica, Facultad de Química, Universidad de la República, Montevideo 11200, Uruguay; qf.mppereira@gmail.com (M.P.P.); juan.gambini@cudim.org (J.P.G.); pablo.duarte@cudim.org (P.D.); javier.giglio@cudim.org (J.G.G.); 2 Centro Uruguayo de Imagenología Molecular, CUDIM, Montevideo 11600, Uruguay

**Keywords:** ^18^F labeling, breast cancer imaging, click chemistry

## Abstract

“Click reactions” are a very useful tool for the selective conjugation of different molecular subunits to produce complex structures in a simple way. In this paper, we present the application of Cu(I)-catalyzed biorthogonal reactions between alkynes and azides to the indirect radiofluorination of an estradiol derivative with potential applications in estrogen receptor imaging. The procedure was fully developed on an automated synthesis platform, and conditions were optimized to achieve the desired product with a reasonable yield without precipitation. Although the biological results were not adequate for a potential radiopharmaceutical, the outcome of this work is valuable since the use of automated platforms is required for the reliable and reproducible preparation of PET radiopharmaceuticals in GMP conditions while limiting the radiation dose rates to the personnel.

## 1. Introduction

**Figure 1:**
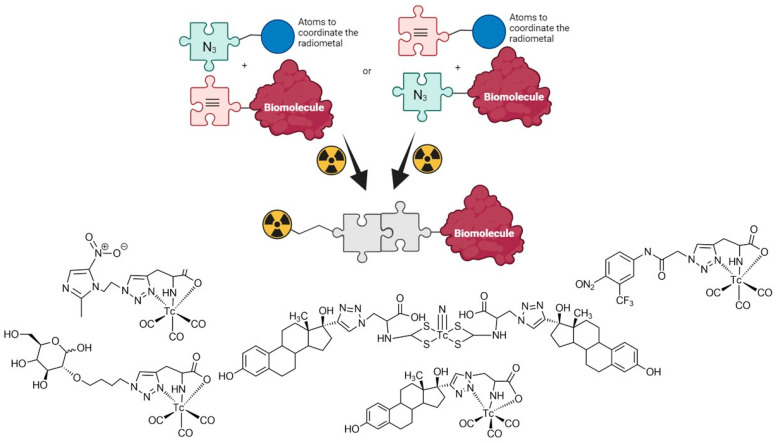
^99m^Tc complexes with different biomolecules synthesized by our group using “click reactions”.

The term “click chemistry” describes a group of reactions characterized by a high yield and high selectivity, thus allowing the joining of 2 molecular units with easily removable byproducts and using mild conditions. This type of reaction was first proposed by Dr. Barry Sharples’s group [1] and since then has been used in a variety of fields, including drug discovery, bioconjugation, materials science, and nanoscience [2,3,4]. The development of radiopharmaceuticals is not an exception [5,6]. The first reported application of “click chemistry” in this field was the assembly of a triazole-based multidentate chelating system attached to biomolecules through the Huisgen 1,3-dipolar cycloaddition of alkynes and azides [7]. In fact, 1,4-functionalized 1,2,3-triazoles are potentially versatile ligands offering several donor sites for metal coordination and have been extensively applied to the preparation of potential ^99m^Tc radiopharmaceuticals [8,9,10]. Our group has utilized this method for the synthesis of glucose, nitroimidazole, flutamide, and estradiol derivatives for technetium labeling (Figure 1) [11,12,13,14,15,16]. 

Another important application of “click reactions” in radiopharmaceutical chemistry is the synthesis of precursors for radiolabeling via the installation of prosthetic groups for radiohalogenation [17,18]. Fluorine-18 is the most frequently used radionuclide in positron emission tomography. Besides its favorable nuclear properties for Molecular Imaging (109.7 min half-life, 635 keV positron energy), the versatile chemistry, including nucleophilic and electrophilic substitutions, allows the direct introduction of ^18^F into diverse molecules of interest. However, the reaction conditions required by direct substitution are not compatible with many biomolecules. On the other hand, indirect methods involving the prior radiosynthesis of a prosthetic group and subsequent bioconjugation have advantages for labeling labile biomolecules since they avoid the harsh reaction conditions of pH or temperature required by direct substitution methods [19,20].

**Figure 2:**
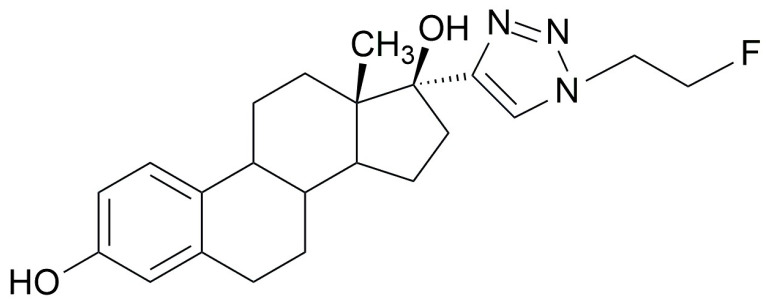
Structure of [^18^F]F-FEET.

The present work describes the application of the Huisgen cycloaddition to the synthesis of [^18^F]F-FEET (Figure 2), an estradiol derivative with potential applications in molecular imaging of the estrogen receptor (ER). The estrogen receptor (ER) is a nuclear receptor that plays a very important role in breast cancer. ER expression is found in only 6–10% of normal breast epithelial cells, while overexpression of this receptor exists in 70% of primary breast cancers. Estrogen receptor-positive (ER+) breast tumors generally respond to anti-estrogen hormone therapy, while ER-negative (ER−) tumors require other types of treatment [21,22,23,24]. The accurate determination of the presence of the ER in breast tumors is very important as a prognostic factor and allows a more accurate prediction of the response to hormonal therapy. One of the tools used to study the expression of this receptor is a biopsy. However, tumors are generally heterogeneous; therefore, a small tissue sample may not represent the exact biology of the tumor. Furthermore, biopsies are invasive, carry some inherent risks, can be painful, can disrupt the tumor microenvironment, and can miss important parts of the tumor [23,24]. Molecular Imaging through Nuclear Medicine is certainly complementary to a biopsy in this field and also presents many advantages; it is minimally invasive, allows data to be collected over time, provides functional information, and covers large volumes of tissue (the whole body in most cases) [25]. Since 1980, many research groups have evaluated steroidal and non-steroidal compounds such as PET and SPECT radiotracers for ER imaging in patients with breast cancer. However, most of them have failed in preclinical studies or in the early stages of clinical ones. In 1984, Kiesewetter et al. [26] developed ^18^F-Fluoroestradiol ([^18^F]FES) [21,26,27]. Various studies have shown that [^18^F]FES has good sensitivity to detect uptake in primary and metastatic tumors and that it is a very useful tool for monitoring hormonal therapy. However, [^18^F]FES has some limitations since liver metastases cannot be detected with this method since they are hidden due to the normal liver metabolism of the tracer [22,25,28,29,30,31]. In May 2020, the FDA approved its use as an imaging agent in PET, indicated for the detection of ER+ lesions and as a complement to a biopsy in patients with recurrent breast cancer [32]. Despite this, the development of tracers aimed at the ER that improve the limitations presented by [^18^F]FES continues to be desirable.

[^18^F]F-FEET (13S,17S)-17-(1-([^18^F]2-fluoroethyl)-1H-1,2,3-triazol-4-yl)-13-methyl-7,8,9,11,12,13,14,15,16,17-decahydro-6H-cyclopentaphenanthrene-3,17-diol has the commonly accepted pharmacophore presentation for an ER agonist, including the presence of an aromatic ring together with groups capable of forming hydrogen bonds (the OH in C3 and the OH in C17). There is a precise distance of 11Å between the oxygen atoms of the OH groups attached to C3 and C17 and a rigid hydrophobic skeleton; all these things are necessary to preserve the biological activity [33,34]. Fluorination was achieved by an indirect method incorporating in position 17 of ethinylestradiol a prosthetic group consisting of a triazole ring (coming from the “click reaction”), which has a 2-carbon chain and the [^18^F]F incorporated in the 4-position of the triazole (Figure 2).

## 2. Results

**Figure 3:**
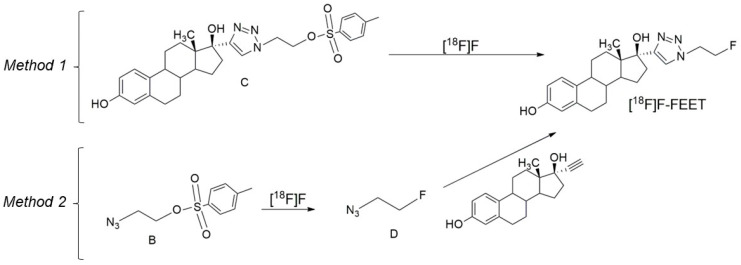
Synthesis of [^18^F]F-FEET by methods 1 and 2.

The synthesis of the radiotracer [^18^F]F-FEET was carried out through two methods. Method 1 starts from a precursor C that contains the estradiol backbone and a tosyl group as a leaving group required for the nucleophilic substitution with fluor. Method 2 starts from a prosthetic group B that contains tosylate as a leaving group and an azide group that was used to incorporate the pharmacophore under milder conditions through a Huisge reaction with ethinylestradiol. Both methods are shown in Figure 3.

The non-radioactive FEET ([^19^F]F-FEET) was also synthesized to confirm the identity of the radiolabeled analog through coinjection in high-performance liquid chromatography (HPLC). [^19^F]FEET was synthesized by incorporating the fluorine atom directly onto compound C using tetrabutylammonium fluoride in DMF by a standard procedure described in the literature [35]. The product was purified, and the structure was confirmed by spectroscopic methods.

### 2.1. Synthesis of [^18^F]F-FEET- Method 1

#### 2.1.1. Synthesis of C (Precursor)

**Figure 4:**
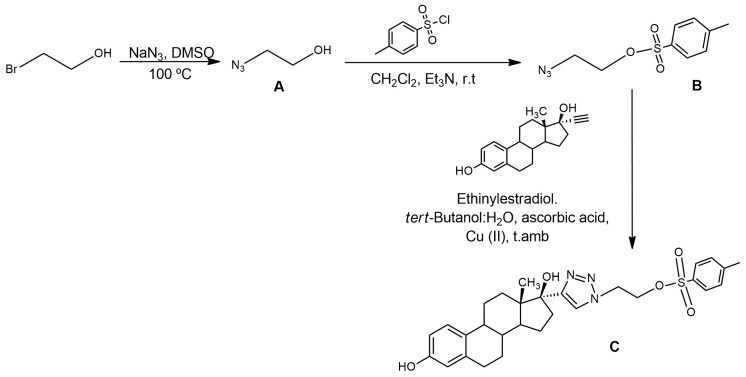
Synthesis of precursor (C).

The synthesis of 2-(4-((13S,17S)-3,17-dihydroxy-13-methyl-7,8,9,11,12,13,14,15,16,17-decahydro-6H-cyclopenta[a]phenanthren-17-yl)-1H-1,2,3-triazol-1-yl)ethyl 4-methylbenzenesulfonate (C) was performed by a two steps] procedure using 2-bromoethanol as the starting reagent as shown in Figure 4. The first reaction step involved the substitution of the bromine atom for an azide group to obtain A. The second step was the incorporation of the tosyl group as a good leaving group to obtain B, and finally, a Huisgen reaction between the azide of B and the terminal triple bond of ethinylestradiol to obtain C (Figure 4).

The substitution of the bromine atom for the azide group was achieved using sodium azide in anhydrous dimethyl sulfoxide for 24 h through the technique of Berta et al. [36], obtaining A with a yield higher than 90%. The incorporation of the tosyl group was carried out according to the technique of Hay et al. [37]. After five days of reaction, although the starting reagent methylsulfonyl chloride was not completely consumed, the reaction was finalized due to the presence of a new species with a lower Rf, verified by chromatographic analysis. This species was purified by column chromatography, using alumina as the stationary phase, obtaining B with a yield of 17%. The last step was the “click reaction,” a 3 + 2 cycloaddition catalyzed by Cu(I). The terminal alkyne of ethinylestradiol reacted with the azide of B to form a triazole ring. This reaction occurred in an aqueous medium and was catalyzed by Cu(I). Cu (I) was generated in situ by the action of ascorbic acid in the presence of Cu(II) [7,13,14]. After 6 days of reaction, extractions with ethyl acetate were carried out, and C was obtained with a yield higher than 100% due to the presence of residual ethinylestradiol and salts in the crude reaction.

#### 2.1.2. Radiofluorination of C to Obtain [^18^F]F-FEET

**Figure 5:**
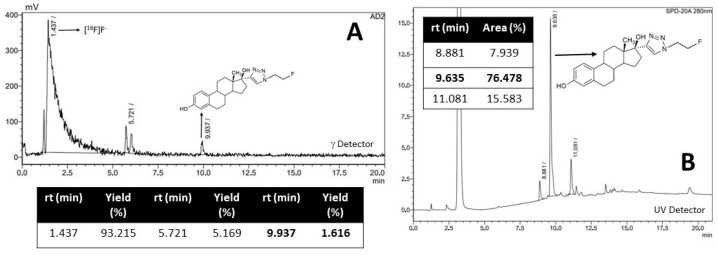
(**A**) γ chromatographic profile obtained in the radiofluorination of C, (**B**) UV chromatographic profile obtained with [^19^F]F-FEET.

The radiofluorination of C with ^18^F was pursued by nucleophilic substitution on a Synthra RNplus Research automated synthesis module. The procedure consists of the incubation of precursor C dissolved in DMSO, using Kryptofix^®^ (Sigma Aldricht, San Luis, MI, USA) as a phase transfer catalyst in combination with K_2_CO_3_ to give a basic medium at 50 °C for 30 min [35,38]. The reaction was monitored by HPLC using a gamma detector. The gamma chromatographic profile demonstrated the presence of a large amount of unreacted fluorine (retention time of 1.4 min) and a species with a retention time of 9.9 min and 1.6% (not decay corrected (ndc)) of a reaction yield that corresponds to [^18^F]F-FEET (Figure 5A), as demonstrated by coinjection with [^19^F]F-FEET (Figure 5B).

**Figure 6:**
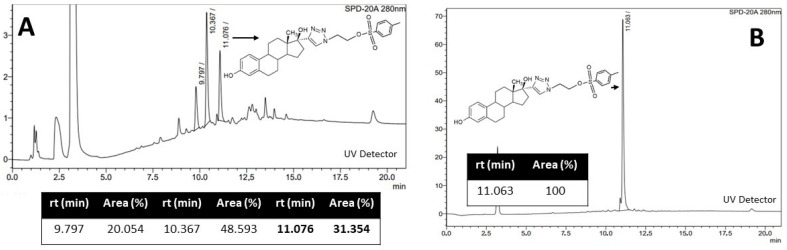
(**A**) UV chromatographic profile obtained in the radiofluorination of C, (**B**) UV chromatographic profile of purified C.

On the other hand, in the UV chromatographic profile at 280 nm of the radiofluorination of C (Figure 6A), the decomposition of C could be observed since several species appeared; only one of them coincided with the retention time of C (11 min) (Figure 6B). We concluded that the low yield of the reaction was a consequence of the decomposition of the precursor compound C due to the harsh conditions required by the direct method of radiofluorination and consequently decided to switch to method 2.

### 2.2. Synthesis of [^18^F]F-FEET- Method 2

#### 2.2.1. Radiofluorination of B to Obtain D

The second method for the synthesis of [^18^F]F-FEET was the nucleophilic substitution of 2-azidoethyl 4-methylbenzenesulfonate (B), which has a tosylate as leaving group to obtain 1-azido-2-[^18^F]fluoroethane (D). Additionally, B has an azide group to incorporate the biomolecule by a Huisgen reaction as a second step (Figure 3—Method 2).

The radiofluorination of B with [^18^F]fluoride was performed on the Synthra RNplus Research automated synthesis module under conditions selected based on the literature and the previous experience of our group [35,38]. The reaction was performed at 90 °C for 20 min, using Kryptofix (Sigma Aldricht, San Luis, MI, USA) and K_2_CO_3_. The mass of B required to achieve a yield greater than 90% (ndc) was 2 mg, since by decreasing the mass from B to 1 mg, the yield decreased considerably (69%) (ndc).

#### 2.2.2. Manual Synthesis of [^18^F]F-FEET

Once the fluorinated precursor D was obtained (Section 2.2.1) with an adequate yield (˃90%, ndc), the ethinylestradiol was coupled using the “click reaction” to obtain the radiotracer of interest. The initial conditions were selected based on the literature (Hausner et al. [39], Marik et al. [40], and Evans et al. [41]). 

Initially, a 10:1 ethinylestradiol/D molar ratio, a 3:1 ethinylestradiol/CuI (catalyst) molar ratio, and a significant excess of sodium ascorbate and base were used. In 10 min at room temperature, the species of interest was obtained with a yield of 31% (ndc). By increasing the reaction time without varying the temperature, the yield increased up to 99% (ndc) (Table 1 entries 1, 2, and 3). Under these conditions, the presence of a precipitate was observed, which prevented automation. Consequently, the mass of ethinylestradiol was reduced, as it is the most insoluble reagent, and the reaction temperature was increased to favor its dissolution. When a 5:1 ethinylestradiol/D molar ratio was used, and the equivalents of the rest of the reagents were maintained (15 min at 80 °C), a yield of 69% (ndc) was obtained (Table 1 entry 4). An increase in the reaction time to 30 min resulted in a yield of 94%, ndc (Table 1 nº5). Unfortunately, the presence of a precipitate was again observed. 

The reaction was also performed by increasing the equivalents of D and reducing the equivalents of Cu (I), sodium ascorbate, and the base (Table 1, entries 6, 7, and 8). A good yield was achieved (Table 1, entry 6). but precipitation occurred in all cases. 

Although the conditions for good fluorination yield were achieved, the presence of a precipitate led to the decision to use more soluble reagents, in accordance with the report by Glaser et al. [42], who uses Cu(II) in the form of CuSO_4_. Methanol was selected as a solvent; the reaction temperature was 80 °C in all cases, while the equivalents of the reagents and the reaction times were varied (Table 2). Initially, a 6:1 ethinylestradiol/D molar ratio, 9 eq. of CuSO_4_, 30 eq. of sodium ascorbate, and a reaction time of 15 min were assayed, obtaining a yield of 6%, ndc (Table 2, entry 1). The equivalents of CuSO_4_ and sodium ascorbate were increased to 30 and 300, respectively, and the mass of ethinylestradiol and D remained constant (Table 2, entry 2). In these conditions, the yield increased to 55%, ndc. A further increase in the reaction time to 30 min improved the yield to 85% (ndc) (Table 2, entry 3). Although using the last conditions, a precipitate was also observed; it was smaller than the one with the previous synthetic strategy. The best result (yield 41%, ndc; Table 2, entry 9) was obtained when using twice the equivalents of ethinylestradiol in relation to the fluorinated compound: 30 equivalents of CuSO_4_, 100 equivalents of sodium ascorbate, and 30 min at 80 °C. 

**Figure 7:**
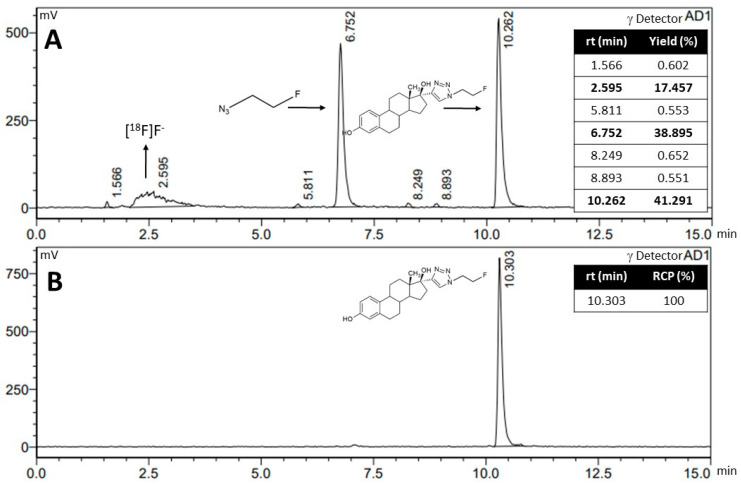
(**A**) Chromatographic profile obtained for entry 9 of Table 2; (**B**) Chromatographic profile obtained after the purification of [^18^F]F-FEET.

Figure 7A shows the chromatographic profile obtained for the reaction conditions corresponding to entry 9 of Table 2. A main species with a retention time of 10.3 min, corresponding to [^18^F]F-FEET, an impurity at 6.7 min, corresponding to the unreacted D, and fluoride with a 2.6 min retention time were observed. The purification of the product of interest was performed by solid phase extraction using a Sep-Pak^®^ (Waters, Germany) C18 Plus light cartridge, obtaining a single species with radiochemical purity (RCP) > 90% and a retention time of 10.3 min (Figure 7B).

#### 2.2.3. Complete Synthesis of [^18^F]F-FEET in an Automated Module

**Figure 8:**
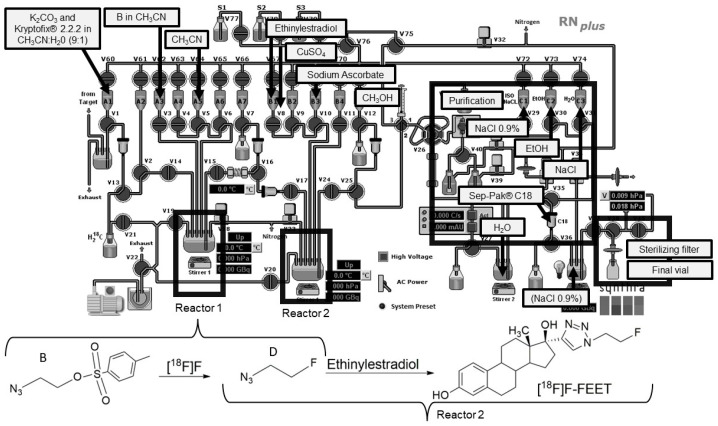
Complete synthesis of [^18^F]F-FEET in an automated module Synthra RNplus.

[^18^F]F-FEET was fully synthesized in the Synthra RNplus Research module with a synthesis time of 80 min and an activity of 3330–3700 MBq. As shown in Figure 8, the synthesis starts from B, and the tosyl group is replaced by radioactive fluorine in reactor 1 of the module, obtaining D. The solution from reactor one is transferred to reactor two, where the Huisgen reaction occurs to obtain [^18^F]F-FEET. Final purification was performed by solid phase extraction using a Sep-Pak^®^ C18 Plus light cartridge Waters, Germany, also in the module. The purified product was filtered through a sterilizing filter, and the radiochemical purity was controlled by HPLC, showing in all cases a single species with a retention time of 10.3 min and 100% RCP.

### 2.3. Physicochemical Studies

Physicochemical studies, including stability in the labeling milieu, stability in human serum, lipophilicity, and plasma protein binding (PPB), were carried out for the purified [^18^F]F-FEET (Table 3) [13].

The stability in the labeling milieu was evaluated by determining the variation of the RCP with time by HPLC up to 4 h after labeling. The RCP was higher than 95% throughout the evaluated period. The stability in human plasma was studied by incubating the purified radiotracer with human plasma at 37 °C and monitoring the RCP (after precipitating the proteins) at different times by HPLC. In this way, only the stability of the free fraction of the tracer can be determined. The [^18^F]F-FEET was stable (RCP greater than 95%) in plasma for up to 4 h of study.

The lipophilicity was determined through the partition coefficient between a 0.1 M phosphate buffer pH = 7.4 and octanol, resulting in a value of 1.8 ± 0.1 PPB, which, determined by size exclusion chromatography, was 58 ± 7%.

### 2.4. In Vitro Biological Studies

The in vitro studies were carried out using the MCF-7 line (ATCC^®^ HTB-22TM, Manassas, VA, USA) [43].

The uptake of [^18^F]F-FEET by MCF-7 cells was determined at different activity values of the tracer (0.37, 0.74, 1.85, 3.7 MBq) incubated for 1 h at 37 °C and in 5% CO_2_. The results are shown in Table 4. 

The variation in the uptake with the incubation time (30, 60, and 120 min) was also determined using 0.74 MBq of the radiotracer (Table 5). The results shown in both tables demonstrate that uptake is almost independent of the activity of the radiotracer and the incubation time in the studied ranges [13,14,44]. 

## 3. Discussion

The synthesis of the estradiol derivative [^18^F]F-FEET via “click chemistry” was pursued by nucleophilic substitution using both direct and indirect fluorination methods. The first strategy was a classic approach, in which the radiofluorination was directly performed on the previously synthesized precursor C, which has the estradiol backbone and a tosylate, a good leaving group required for the nucleophilic reaction with ^18^F. 

Precursor C was previously prepared in 3 steps. The first step involved the substitution of the bromine atom of the commercial reagent 2-bromoethanol with the azide group of ethinylestradiol using NaN_3_ in DMSO. The second step was the incorporation of the tosyl group using methylsulfonyl chloride in a basic medium and CH_2_Cl_2_ as solvent. Finally, a Huisgen reaction between the azide of B and the terminal triple bond of ethinylestradiol was performed. The structure of all the intermediate products was confirmed by proton NMR. However, the proton NMR of C showed the presence of a small amount of the starting reagent ethinylestradiol as an impurity. The identity of C was confirmed by a signal as a singulet at δ = 7.52 ppm, which does not appear in the spectrum of ethinylestradiol and corresponds to the H of the triazole ring according to the literature and the experience of our group [13,14], thus confirming the success of the “click reaction.” In addition, the NMR analysis showed the presence of the rest of the protons of C.

C was radiofluorinated by direct nucleophilic substitution at 90 °C, but the product [^18^F]F-FEET was obtained with a very low yield. After confirming that, due to the drastic radiofluorination conditions, C was decomposed, yielding various unidentified degradation products, it was necessary to find an alternative method for the incorporation of ^18^F into the estradiol backbone without affecting its structural integrity.

Typically, the radionuclide is incorporated into biomolecules during the final step of synthesis. However, if the biomolecule is labile, the incorporation of the radionuclide in a small molecule (prosthetic group) is recommended. The prosthetic group should have an appropriate structure that allows the posterior conjugation with the biomolecule by an easy procedure using mild conditions [5,6]. 

Many prosthetic groups for this type of reaction are reported in the literature, but the ones bearing either an azide or a triple bond are especially convenient due to the reliability and selectivity of “click reactions”. In addition, the robustness and modularity of the “click reactions” are particularly attractive for automated processes usually applied for procedures using positron emitters [5,6,45]. The automated synthesis modules grant the preparation of radiopharmaceuticals in a reliable and reproducible way while limiting the radiation dose rates to the personnel and, when necessary, allow working under good manufacturing practices (GMP). However, the use of insoluble products in organic polar solvents is difficult to implement since the modules contain tubing with a low caliber and valves, which are easily clogged by precipitates. The challenge to adapt a “click reaction” to an automatic platform is to find the conditions for a good balance between an adequate yield and without precipitation of the reagents.

Although an important number of references for “click reactions” using ^18^F in automated modules can be found in the literature, in most cases, automation was only used for the synthesis of the [^18^F]F-labeled prosthetic group, while the “click reaction” to incorporate the biomolecule was performed manually [6].

This paper, on the other hand, presents an optimized method to perform the synthesis of the radiotracer of interest by a fully automated procedure. To achieve this goal, the “click reaction” between the prosthetic group and ethinylestradiol was first optimized manually. 

“Click reactions” usually have two paths to follow: either the use of CuI as a catalyst or Cu(II) salts in an aqueous medium together with sodium ascorbate to generate Cu(I) in situ. In the present research, we began working with the first strategy, finding conditions that generate the product with a good yield but without the presence of a precipitate, which prevents automation. Consequently, we turned to the second path using more soluble reagents, like CuSO_4_, and adding methanol to dissolve the ethinylestradiol at a temperature of 80 °C. In this case, an overall yield of 41% without precipitate was achieved.

The optimized conditions were transferred to the complete synthesis of [^18^F]FEET in a Synthra RNplus Research module, maintaining the achieved yield.

There are only a few works in the literature that perform fully automated “click chemistry” reactions for radiofluorinations. One of them is the work by Iannone et al. [46], who incorporated ^18^F into a different prosthetic group and then used a “click reaction” to couple it to a new PSMA-617 derivative using Cu (II) salts and sodium ascorbate. Iannone’s group carried out exhaustive optimization since their biomolecule was very insoluble, finding the best conditions by using a mixture of aqueous and organic solvents to avoid precipitation as we did. The final yield was only 6.1% ndc, much lower than the one achieved in the present work.

The second example is by Zhang et al. [47], who developed [^18^F]DHMT for reactive oxygen species imaging. In this case, ^18^F was incorporated into the same prosthetic group used in this work (1-azido-2-[^18^F]fluoroethane) but with a lower yield (71–87%, ndc) compared to ours (>90%). The “click reaction” was carried out using a commercial Cu(I)-stabilizing ligand, tris[(1-benzyl-1H-1,2,3-triazol-4-yl)methyl]amine (TBTA), in a mixture of aqueous and organic solvents, obtaining a yield of 6.9% (ndc) of the final product, less than the yield obtained in our work (40%). These results show the difficulties of carrying out this type of reaction on automatic synthesis platforms and the need to continue working to optimize processes with the purpose of facilitating the use of this type of reaction for the synthesis of radiotracers for routine clinic applications.

Once the [^18^F]F-FEET was obtained, physicochemical studies, stability studies, and in vitro biological studies were carried out. The radiotracer was stable in the reaction milieu and in human plasma for at least 4 h, has a lipophilicity in the adequate range to cross biological membranes [48,49], and has a relatively high PPB. These physicochemical parameters are adequate as a potential radiopharmaceutical for ER imaging. The ER is a nuclear receptor and, consequently, a radiotracer without a specific transporter to cross the cell membrane needs to penetrate by passive diffusion. The PPB, on the other hand, is a very important parameter that determines the pharmacokinetics of the potential radiopharmaceutical. In general, blood clearance is slower for compounds with a high PPB. [^18^F]F-FEET has a high PPB of 58 ± 7 but is significantly lower than the starting biomolecule, ethinylestradiol, which has a PPB of 98.3 ± 0.6 according to the literature [50].

The biological in vitro studies were carried out in the MCF-7 cell line [43]. The popularity of the MCF-7 cell line for breast cancer research reflects its fidelity in many clinical aspects of this disease, particularly ER+ breast cancer. One of the most important contributions of the MCF-7 cell line has been in the study of ERα since it is one of the few lines to express substantial levels of it, mimicking the majority of invasive breast cancers [51].

As shown in Table 3 and Table 4, the % uptake was low and independent of the activity of the tracer and the incubation time. The uptake of [^18^F]F-FEET (0.8 ± 0.2%) is very low compared to natural estradiol (uptake determined under the same experimental conditions 6.6 ± 1.4%) and with ^18^F-FES (uptake determined under the same experimental conditions 6.3 ± 1.3%), the gold standard for the molecular imaging of estrogen receptors [14]. From this result, we can conclude that the structural changes introduced by the derivatization used to couple the radionuclide interfered with uptake by the receptor. Furthermore, a similar estradiol derivative, [^18^F]F-FETE, developed by Xu et al. [52], also suffered from a low uptake in MCF-7 cells. In both radiotracers, there is a triazole ring in position 17 of estradiol. [^18^F]F-FEET has a 2-carbon chain in the 4-position of the triazole, while [^18^F]F-FETE has a 6-carbon chain and two oxygens. The conclusion is that although estradiol was derivatized in position 17, which, according to the literature, is not crucial for biological activity, the sensitivity of ER receptors to structure is very high, making it very difficult to develop this type of radiotracer. This result is in accordance with the fact that many unsuccessful attempts have been made to develop ER imaging agents, and even ^18^F-Fluoroestradiol ([^18^F]FES), which has been approved by the FDA [32] for breast cancer imaging and has disadvantages like high hepatobiliary elimination [22,25,28,29,30,31].

## 4. Materials and Methods

### 4.1. General

All chemicals were reagent grade and were used without further purification. Thin-layer chromatography (TLC) was performed on percolated silica gel plates (Sigma Aldrich, Merck Group, Sant Luis, MI, USA). The developer used commercial anisaldehyde. ^1^H Nuclear magnetic resonance (NMR) was obtained at 400 MHz in dichloromethane. The chemical shifts, *δ*, are reported in ppm (parts per million) relative to residual solvent peaks. The multiplicity in the ^1^H NMR is defined by s (singlet), d (doublet), t (triplet), or m (multiplet). Solvents for synthesis were anhydrous grade, and solvents for chromatographic analysis were HPLC grade. Analytical chromatography was performed with a Shimadzu HPLC-UFLC Prominence HPLC, coupled to a diode array UV detector SPD-M20A (Shimadzu, Japan) and a solid scintillation detector B-FC-3200 (LAB LOGIC). The chromatographic system was as follows: Phenomenex Phenosphere ODS 80 Å, 250 × 4.6 mm, and a 5 μm column using A-water and B-acetonitrile as solvents. The gradient was from 0 to 10 min from 10% to 90% B and from 10 to 15 min 90% B at flow 1.5 mL/min using UV detection at 280 nm and gamma detection. Chromatographic data were acquired and analyzed with LabSolutions^®^, version 5.9. Radioactivity measurements were performed using a Capintec CRC 25 dose calibrator. Physicochemical studies and in vitro studies were measured in a solid scintillation counter Multichannel ORTEC (digiBASE) solid scintillation (1086 channels) with crystal: Na(Tl)I diameter 2.5 inch × 2.5 inch.

### 4.2. Synthesis

#### 4.2.1. Synthesis of A

A mixture of 2-bromoethanol (1.34 g–10.95 mmol) and sodium azide (NaN_3_) (0.85 g–13.14 mmol) in anhydrous dimethyl sulfoxide (DMSO) (30mL) was incubated with continuous shaking and in a nitrogen atmosphere at 100 °C for 6 h and a further 18 h at room temperature. After the reaction time had elapsed, the solvents were removed under reduced pressure at 60 °C, and extractions were carried out with ether and water (10 × 10 mL). The organic phase was dried with anhydrous magnesium sulfate, and the solvents were removed under reduced pressure at 25 °C to obtain A (yellow oil, yield = 90%).

NMR A ^1^H (400 MHz, (CDCl_3_) δ (ppm): 3.76 (m, 2H), 3.38 (t, 2H).

#### 4.2.2. Synthesis of B

The reaction crude of A (0.87 g–9.99 mmol) in anhydrous dichloromethane (CH_2_Cl_2_) (30 mL) and triethylamine (Et_3_N) (2 mL–6.46 mmol) was cooled to 0 °C. A solution of methylsulfonyl chloride dissolved in CH_2_Cl_2_ (2.26 g–11.90 mmol) was added dropwise. Subsequently, the reaction was removed from the ice bath and kept at room temperature, with continuous stirring and in a nitrogen atmosphere for 5 days. The purification of B was performed by column chromatography using alumina and a mixed solvent consisting of 7:3 hexane/ethyl acetate. (yellow oil, yield = 17%).

NMR B ^1^H (400 MHz, (CDCl_3_) δ (ppm): 7.83 (d, 2H), 7.38 (d, 2H), 4.17 (t, 2H), 3.49 (t, 2H), 2.46 (s, 3H).

#### 4.2.3. Synthesis of C

B (0.66 g–2.73 mmol) and ethinylestradiol (0.40 g–1.36 mmol) were incubated in tert-butanol (6 mL) together with ascorbic acid (0.011 g–0.055 mmol) and copper acetate (0.020 g–0.11 mmol) dissolved in water (4 mL). The mixture was left to react for 6 days at room temperature, protected from light and under a nitrogen atmosphere. Once the reaction was completed, it was extracted with ethyl acetate (10 × 10 mL). The organic phase was dried with anhydrous magnesium, and the solvents were removed under reduced pressure at 40 °C (yellow oil, yield > 100%).

NMR C ^1^H (400 MHz, (CDCl_3_) δ (ppm): 7.69 (d, 2H), 7.52 (s, 1H), 7.31(d, 2H), 7.17 (d, 1H), 7.06 (d, 1H), 6.65 (dd, 1H), 6.62 (dd, 1H), 6.57 (d, 1H), 6.55 (d, 1H), 4.66 (t, 2H), 4.42 (t, 2H), 2.80 (m, 4H), 2.61 (s, 1H), 2.40-1.25 (m, 20H), 1.05 (s, 3H), 0.88 (s, 6H).

#### 4.2.4. Synthesis of [^19^F]F-FEET

C (0.05 g–0.09 mmol) was dissolved in anhydrous dimethylformamide (DMF) (2.5 mL) and tetrabutylammonium fluoride (TBAF) (0.030 g–0.11 mmol) for 6 h at 130 °C, protected from light and under a nitrogen atmosphere. After this time, extractions with ether and H_2_O (10 × 10 mL) were performed. The solvents were removed under reduced pressure at 60 °C (yellow oil, yield = 39.6%). MS (EI, 10 eV) *m*/*z*: 385 (M+).

### 4.3. Radiolabeling

#### 4.3.1. Production of [^18^F]F^−^

[^18^O]H_2_O (enrichment > 98%) (Huayi isotopes) was placed in a fluorine-18 PETtrace target (General Electric) and was irradiated with 16 MeV protons to produce approximately 18.5–55.5 GBq (18,500–55,500 MBq) of [^18^F]F^−^ via the ^18^O(p,n)^18^F. The [^18^F]F^−^ was transferred to a Synthra RN plus Research module.

#### 4.3.2. Conditioning of ^18^F

The [^18^F]F^−^ obtained as an aqueous solution in [^18^O]H_2_O was transferred to a Synthra RN plus Research module and purified using a Sep-Pak^®^ QMA Light (Waters, Germany) ion exchange cartridge. [^18^F]F^−^ was eluted from QMA to the reactor with a solution containing K_2_CO_3_ (3.5 mg) and Kryptofix 2.2.2 (Sigma Aldricht, San Luis, MI, USA) (15 mg) in H_2_O/MeCN (1:9, 1000 μL). H_2_O was removed under reduced pressure with a He(g) stream and heating to 95 °C.

#### 4.3.3. Radiofluorination of C to Obtain [^18^F]F-FEET

Once the conditioned [^18^F]F^−^ was in the reactor, a solution of C (1 mg–0.002 mmoles) in dry MeCN (1 mL) was incorporated. Radiofluorination was carried out for 20 min at 90 °C.

#### 4.3.4. Radiofluorination of B to Obtain D

The conditioned [^18^F]F^−^ (1850–5550 MBq) was mixed with a solution of B (2 mg–0.008 mmoles) in dry MeCN (1 mL). Radiofluorination was carried out for 20 min at 90 °C. 

#### 4.3.5. Complete Synthesis of [^18^F]F-FEET in an Automated Module

The conditioned [^18^F]F^−^ (3330–3700 MBq) was mixed in the reactor with a solution of B (2 mg–0.008 mmoles) in dry MeCN (1 mL). Radiofluorination was carried out for 20 min at 90 °C. After heating was completed, the mixture was transferred to the second reactor where the Huisgen reaction was carried out with ethinylestradiol (0.001 mmol–2eq), a 0.45 M copper sulfate (CuSO_4_) solution (0.01 mmol–30 eq), a 1.5 M sodium ascorbate (0.05 mmol–100 eq) solution in 300 µL of methanol, and 100 µL of 50 mM pH = 6 phosphate buffer at 80 °C for 30 min. Purification was performed by solid phase extraction using a Sep-Pak^®^ C18 Plus (Waters, Germany) light cartridge. Specific Activity = 0.22–0.25 GBq/µmol.

### 4.4. Physicochemical Studies

#### 4.4.1. Stability in the Labeling Milieu

The tracer was incubated at room temperature for a period of 4 h. Samples was extracted at 1, 2, 3, and 4 h post-labeling, and radiochemical purity was determined using the HPLC conditions detailed in Section 4.1.

#### 4.4.2. Stability in Human Plasma

The tracer (100 µL, 47–53 MBq) was incubated in human plasma (1000 µL) at 37 °C. After 1, 2, 3, and 4 h, 200 µL samples were extracted, and proteins were precipitated by adding cold absolute ethanol (200 µL, −15 °C), followed by incubation at −20 °C for 5 min. The sample was centrifuged at 100× *g* (5 min, 0 °C), and the radiochemical purity of the supernatant was monitored by HPLC, using the conditions described in Section 4.1. 

#### 4.4.3. Lipophilicity

Lipophilicity was studied at pH 7.4 by determining the 0.1 M octanol/buffer phosphate partition coefficient (log Po/aq). In a centrifuge tube, 2 mL of octanol and 1.9 mL of 0.1 mL of phosphate buffer (pH = 7.4) were mixed. The tracer (100 μL, 47–53 MBq) was added, and the mixture was shaken in a vortex for 2 min, centrifuged for 5 min at 4000 rpm, and 2 aliquots of each phase (100 μL) were extracted. The activity of each of them was measured on a solid scintillation counter. Three repetitions of each experience were performed. The distribution coefficient was calculated as follows: log P o/aq = (counts in organic phase/counts in aqueous phase).

#### 4.4.4. Plasma Protein Binding

Plasma protein binding (PPB) was determined by size exclusion using Illustra MicroSpin G-50 columns (GE Healthcare, Chicago, IL, USA). A blank (475 μL of distilled water + 25 μL of the tracer) and a sample (475 μL of human plasma + 25 μL 12–14 MBq of the tracer) were incubated at 37 °C for 30 and 60 min. After the stipulated time, the column conservation buffer was extracted by centrifugation for 1 min at 716× *g*, and 25 μL of the blank or sample was seeded on the column and centrifuged for 1 min at 716× *g*. The activity eluted and retained on the column was measured in a solid scintillation counter. The protein-bound fraction was calculated as the percentage of activity eluted from the column.

### 4.5. In Vitro Studies

For the in vitro biological evaluation, the adherent cell line MCF-7 (ATCC^®^ HTB-22TM, Manassas, VA, USA), corresponding to a human mammary adenocarcinoma, was used. Cells were grown in T-75 flasks (Greiner bio-one, Sigma Aldrich, Merck Group, Sant Luis, MI, USA) with a DMEM medium (A1316, 9050 PanReac AppliChem ITW Reagents, Darmstadt, Germany) supplemented with 10% fetal bovine serum (Gibco, TermoFisher Scientific, Waltham, MA, USA)), penicillin 100 U mL^−1^ (Sigma Aldrich, Merck Group, Sant Luis, MI, USA), and streptomycin 100 μg mL^−1^ (Sigma Aldrich, Merck Group, Sant Luis, MI, USA) at 37 °C and 5% CO_2_.

#### 4.5.1. Uptake Assay Dependent on the Activity of the Radiotracer

The cells (monolayer, 1 × 10^6^ cells per T-75 flask) were incubated with [^18^F]F-FEET (0.37, 0.74, 1.85, and 3.7 MBq) for 1 h at 37 °C and 5% CO_2_. After the incubation time had elapsed, the culture medium was removed, and the cells were washed twice with phosphate-buffered saline (PBS) (10 mL) and treated with trypsin-EDTA (3 mL, 5 min of incubation at 37 °C and 5% CO_2_). The activity in the supernatant and the cells was measured in a scintillation counter. Results were expressed as a percentage of the activity bound to cells.

#### 4.5.2. Uptake Assay Dependent on the Time of Incubation

The cells (monolayer, 1 × 10^6^ cells per T-75 flask) were incubated with 0.74 MBq of [^18^F]F-FEET for 30 min, 1 h, and 2 h at 37 °C and 5% CO_2_. After the incubation time had elapsed, the culture medium was removed, and the cells were washed twice with PBS (10 mL) and treated with trypsin-EDTA (3 mL, 5 min of incubation at 37 °C and 5% CO_2_). The activity in the supernatant (12 mL of culture medium + 20 mL of PBS) and in the cells was measured in a solid scintillation counter. Results were expressed as a percentage of the activity bound to cells.

## 5. Conclusions

This paper presents the application of a “click reaction” for the preparation of a potential radiopharmaceutical through radiofluorination. The selected strategy includes the preparation of a radiolabeled prosthetic group followed by the incorporation of the biomolecule of interest by a Huisgen reaction. The procedure was fully developed on an automated synthesis module and achieved a higher yield compared with the few similar examples found in the literature. Although in vitro biological results were not adequate for an estrogen receptor imaging agent, our results constitute an important contribution to the field. The adjustment of a “click reaction” to an automatic platform is very challenging, as demonstrated by the scarce successful examples in the literature. Consequently, our results can be used in the future for the development of new tracers based on labile biomolecules that do not resist the drastic conditions of the incorporated ^18^F atom using usual conditions.

## Figures and Tables

**Table 1:** Optimization of Huisgen reaction’s conditions using CuI as catalyst.

Entry	Reagents (eq)					Time (min)	Temperature	Yield (%)
	D	Ethinylestradiol	Ascorbic Acid	CuI	DIPEA			
1	0.1	1	31	3	31	10	r.t	31.1
2	0.1	1	31	3	31	45	r.t	81.2
3	0.1	1	31	3	31	65	r.t	98.7
4	0.1	0.5	31	3	31	15	80 °C	69.3
5	0.1	0.5	31	3	31	30	80 °C	94.0
6	0.03	1	10	1	10	30	r.t	84.8
7	1	1.6	-	1.2	3.6	20	60 °C	4.2
8	1	1.6	-	1.2	3.6	30	60 °C	3.9

eq = equivalents, r.t = room temperature. Observations: There is precipitation in all the reaction conditions tested.

**Table 2:** Optimization of Huisgen reaction conditions using CuSO_4_.

Entry	Reagents (eq)				Time (min)	Yield (%)
	D	Ethinylestradiol	CuSO_4_ (0.45 M)	Ascorbic Acid (1.5 M)		
1	1	6	9	30	15	5.7
2	1	6	30	300	15	54.8
3	1	6	30	300	30	84.8
4	1	2	30	300	15	35.3
5	1	2	30	300	30	73.0
6	1	2	30	30	15	3.8
7	1	2	30	30	30	6.2
8	1	2	30	100	15	15.2
**9**	**1**	**2**	**30**	**100**	**30**	**41.2**
10	1	2	30	50	15	20.1
11	1	2	30	50	30	41.7

eq = equivalents, Observations: There is precipitate in all the conditions tested, except the conditions in entry 9 (in bold).

**Table 3:** Physicochemical studies of [^18^F]F-FEET.

	[^18^F]F-FEET
Stability in labeling milieu at 4 h	RCP > 95%
Stability in human plasma at 4 h	RCP > 95%
Log P (o/aq)	1.8 ± 0.1
PPB	(58 ± 7)%.

*n* = 3.

**Table 4:** Uptake of [^18^F]F-FEET in MCF-7 cells using different activities of the radiotracer.

(MBq)	% Uptake
0.37	1.0 ± 0.2
0.74	1.3 ± 0.4
1.85	1.3 ± 0.2
3.7	1.0 ± 0.2

*n* = 3/1 h incubation.

**Table 5:** Uptake of [^18^F]F-FEET in MCF-7 cells determined at different incubation times.

	% Uptake
30 min	0.60 ± 0.04
1 h	0.6 ± 0.1
2 h	1.0 ± 0.5

*n* = 3/0.74 MBq.

## Data Availability

All data supporting the findings of the study are available from the corresponding author, upon reasonable request.
